# Restoration and preservation effects of mung bean antioxidant peptides on H_2_O_2_ ‐induced WRL‐68 cells via Keap1‐Nrf2 pathway

**DOI:** 10.1002/fsn3.3638

**Published:** 2023-08-21

**Authors:** Xue Miao, Xin Liu, Hongsheng Chen, Changyuan Wang, Jingjing Diao

**Affiliations:** ^1^ College of Food Science Heilongjiang Bayi Agricultural University Daqing China; ^2^ Mudanjiang Institute of Food and Biotechnology Heilongjiang Bayi Agricultural University Mudanjiang China; ^3^ National Coarse Cereals Engineering Research Center Heilongjiang Bayi Agricultural University Daqing China

**Keywords:** Keap1/Nrf2 signaling pathway, mung bean antioxidant peptides, oxidative stress, WRL‐68 cells

## Abstract

Mung bean antioxidant peptides (MBAPs) were prepared from mung bean protein hydrolysate, and four peptide sequences including Ser‐Asp‐Arg‐Thr‐Gln‐Ala‐Pro‐His (~953 Da), Ser‐His‐Pro‐Gly‐Asp‐Phe‐Thr‐Pro‐Val (~956 Da), Ser‐Asp‐Arg‐Trp‐Phe (~710 Da), and Leu‐Asp‐Arg‐Gln‐Leu (~644 Da) were identified. The effects of MBAPs on the oxidation‐induced normal human liver cell line WRL‐68 were analyzed to determine the mechanism protecting the oxidation‐induced injury. The results showed that the cells were subjected to certain oxidative damage by H_2_O_2_ induction, as evidenced by decreased cell number and viability, overproduction of intracellular ROS, and decreased mitochondrial membrane potential. Compared with the H_2_O_2_‐induced group, the MBAP‐treated oxidation‐induced group exhibited significantly higher cell number and viability, and the intracellular ROS was similar to that of the control group, suggesting that MBAP scavenges excessive intracellular free radicals after acting on the oxidation‐induced cells. Combined with Western blotting results, it was concluded that the MBAP‐treated oxidation‐induced group also significantly promoted the expression of proteins related to the kelch‐like ech‐related protein 1 (Keap1)/ nuclear factor e2‐related factor 2 (Nrf2) signaling pathway, which resulted in an approximately 2‐fold increase in antioxidant enzymes, and a decrease in malondialdehyde content of approximately 55% compared to oxidatively‐induced cells, leading to the recovery of both cell morphology and viability. These results suggest that MBAPs scavenge intracellular free radicals and improve oxidative stress in hepatocytes through the expression of Keap1/Nrf2 pathway‐related protein, thereby reducing oxidative attack on the liver. Therefore, MBAP is applied as a nutritional ingredient in the functional food field, and this study provides a theoretical basis for the high utilization of mung bean proteins.

## Graphical abstract

MBAPs notably improved the morphological changes, viability, and the expression of Kelch‐like ECH‐associated protein 1 (Keap1)/ nuclear factor E2‐related factor 2 (Nrf2) signaling pathway‐related proteins of hydrogen peroxide‐induced hepatocytes. MBAPs inhibited oxidation‐induced intracellular reactive oxygen species production, mitochondrial damage, and decreased malondialdehyde levels, which increased the levels of antioxidant enzymes, such as superoxide dismutase, catalase, and glutathione peroxidase. MBAPs scavenge intracellular free radicals and improve oxidative stress in hepatocytes through the expression of Keap1/Nrf2 pathway‐related protein, thereby reducing oxidative damage in the liver.

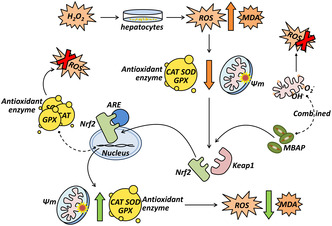

## 1 INTRODUCTION

Mung beans, also known as cowpeas (*Vigna radiate L*.), are regarded as valuable edible legumes in many countries (Yu et al., 2020) due to their purgative and hepatoprotective properties (Li et al., 2021). In addition, mung beans have hypoallergenic properties and are highly nutritious, with a protein content of between 19.7% to 32.1% (Liu et al., 2021). Furthermore, they are rich in amino acids (Ali et al., 2016) and are utilized in many developing countries as a supplement or alternative to meat and milk proteins (Wang, Huang, et al., 2021; Wang, Xing, et al., 2021).

Presently, despite being an excellent protein source, mung bean proteins are a low‐value by‐product of starch or vermicelli processing. The enzymatic hydrolysate of mung bean proteins produces peptide fragments with antioxidant, cholesterol‐lowering, fat‐reducing, and immune‐enhancing properties, making them ideal dietary functionally active components (Diao, 2019). A high‐fat diet can cause elevated levels of free radicals and lipid metabolism disorders in the body. However, mung bean peptides have been reported to improve high‐fat‐induced disorders and significantly increase levels of liver antioxidant enzymes and indicators, such as total cholesterol and triglycerides (Liu, 2022).

Most metabolic illnesses are pathophysiology related to imbalances in producing reactive oxygen species (ROS), resulting in an impaired antioxidant defense system due to aberrant oxidative stress (Barteková et al., 2021). Abnormalities in the body's redox levels can cause abnormalities in lipid metabolism; the organelles involved in the body's metabolism are affected at the oxidative stress level, resulting in phenomena such as lipid peroxidation and mitochondrial dysfunction. Arroyave‐Ospina et al. (2021) demonstrated that oxidative stress contributes to nonalcoholic steatohepatitis and the modulation of antioxidants through the nuclear factor erythroid 2‐related factor 2 (Nrf2) pathway helps the body return to a normal lipid metabolic state. A change in the equilibrium between ROS generation and antioxidant defense has been described as oxidative stress (Betteridge, 2000).

Low ROS levels are required for essential biological activities, such as cell proliferation and differentiation (Bigarella et al., 2014; Theopold, 2009) since they operate as signaling molecules to drive various physiological processes (Ng et al., 2014). Moreover, ROS maintains cellular redox stability to prevent cell expansion and senescence. In contrast, high levels of ROS are harmful and activate a variety of responses and signaling pathways, including the retrograde response (Jiang et al., 2016), the unfolded protein response (Guerra‐Moreno et al., 2019), endoplasmic reticulum stress activation response (Amen et al., 2019), target of rapamycin response (Jazwinski, 2015), and various mitogen‐activated protein kinase pathways (González‐Rubio et al., 2019). These responses and processes require detoxifying enzymes, such as superoxide dismutase (SOD), catalase (CAT), glutathione peroxidase (GPX), and glutathione or glutathione oxygen reductase, and other components, such as thioredoxin, to achieve a dynamic balance between the production and removal of ROS. The present research discovered that food‐derived protein hydrolysates exhibit the ability to balance ROS levels in cells and promote the expression of antioxidant enzymes to protect the body against oxidative damage. Zhang, Zhao, et al. (2022) demonstrated that tuna skin hydrolysate significantly scavenges free radicals in vitro and that it significantly protects human skin fibroblasts (hsfb) from ultraviolet‐a (UVA) damage. Other studies have also confirmed that hydrolysates produced from animal and plant processing by‐products, such as tuna, soybeans, chickpeas, etc., can protect the body against oxidative damage through direct scavenging of free radicals and reduction of free radical production (Juárez‐Chairez et al., 2022; Kong et al., 2023; Sheng et al., 2023).

Mung beans have been utilized for detoxifying and other purposes since ancient times, as documented in the Compendium of Materia Medica and the Kai Bao Ben Cao (Tian et al., 2017). In addition, several studies have confirmed that mung bean protein hydrolysate (MBPH) positively regulates oxidative stress levels. Kusumah et al. (2020) researched the antioxidant potential of a mung bean proteolytic solution by measuring its 2,2′‐Azino‐bis‐(3‐ethylbenzothiazoline‐6‐sulfonate) radical scavenging, ferrous ion chelating ability, and oxygen radical absorption capacity. Xie et al. (2019) and Liu (2022) proved that mung bean protein hydrolysate has a good protective effect on hydrogen peroxide (H_2_O_2_)‐induced NCTC‐1469 cells, and it has a significant promotional effect on hepatic HDL and antioxidant enzyme levels in high‐fat‐induced mice. These results implied that the antioxidant capacity of mung bean protein hydrolysate is associated with the regulation of body metabolism.

The liver, the body's most critical organ for metabolism, is also a primary target tissue for exogenous chemicals and toxicants (Wang, 2022). Therefore, this study aimed to analyze the cell viability and morphology, ROS levels, mitochondrial oxidative damage, and antioxidant enzyme activities of MBAP‐treated oxidation‐induced WRL‐68 cells to further illustrate the mechanism of action of MBAPs in regulating redox homeostasis on oxidized cells and to clarify the mechanism of the effect of its antioxidant capacity on the regulation of hepatic metabolic.

## 2 MATERIALS AND METHODS

### 2.1 Materials

Mung bean protein (80.3%, g/g) was purchased from Zhaoyuan Biological Protein Co., Ltd. The Alcalase® 2.4 L FG protease was purchased from Novozymes A/S. The Sephadex G‐15 column was provided by Shanghai Baoman Co., Ltd. The SOD, CAT, GPX, and malondialdehyde (MDA) assay kits and the cytotoxicity enzyme‐linked immunosorbent assay (ELISA) kit were obtained from the Jiancheng Bioengineering Research Institute Co., Ltd. The DL‐dithiothreitol, iodoacetamide, formic acid, acetonitrile, methanol, and calcein acetoxymethyl ester (Calcein‐AM; CAS 148504‐34‐1) were purchased from Sigma‐Aldrich® (Merck KGaA). The mitochondrial superoxide (MitoSOX™, Cat. number M36008) and the pentahydrate bisbenzimide (Hoechst 32258, 10 mg/mL; Cat. number H3569) stains were from Thermo Fisher Scientific, Inc.

The polyvinylidene difluoride membrane, dihydroethidium (DHE), and antibodies (β‐actin, Kelch‐like ECH‐associated protein 1 [Keap1], Nrf2) were purchased from Beyotime Biotechnology Inc. The inactivated fetal bovine serum, Dulbecco's modified eagle medium, N‐acetyl‐L‐cysteine (NAC), nuclear protein extraction kit, bicinchoninic acid protein quantification kit, and penicillin–streptomycin liquid were obtained from Solarbio Science and Technology Co., Ltd.

### 2.2 Preparation of the mung bean protein hydrolysate

Mung bean protein (7%, w/w) was hydrolyzed using Alcalase 2.4 L. The ratio of the enzyme to the protein substrate was 1:100. The hydrolysis conditions were pH 8.5 at 55°C; hydrolysis was terminated when the degree of hydrolysis reached 25%. The hydrolysate was spray‐dried and stored at room temperature until required.

### 2.3 Isolation and purification of the MBAPs

Subsequently, the MBPH was fractionated utilizing Sephadex G‐15 chromatography according to the method previously described by Diao et al. (2022). The MBPH was diluted in distilled water to a concentration of 100 mg/mL. The solution (0.8 mL) was separated by a Sephadex G‐15 gel column (1 × 40 cm) and eluted with distilled water at a flow rate of 1 mL/min. The elution was monitored by ultraviolet (UV) absorbance at 220 nm. Five peak areas of the MBPH were identified and designated as I, II, III, IV, and V.

### 2.4 Peptide sequence analysis using liquid chromatography–tandem mass spectrometry

The liquid chromatography–tandem mass spectrometry (LC–MS/MS) system consisted of an Easy‐nLC™ 1200 system (Cat. Number LC140) coupled with a Q Exactive™ Hybrid Quadrupole‐Orbitrap™ mass spectrometer (MS) (Cat. number IQLAAEGAAPFALGMBDK; both Thermo Fisher Scientific, Inc.) with a nano‐electrospray ionization source. The chromatography was performed on a C18 reverse‐phase high‐performance liquid chromatography column (1.9 um, 100 Ä, Dr. Maisch GmbH) and separated by developing an acetonitrile gradient in 0.1% formic acid from 5% to 65% over 15 min. The eluate was fed directly into the Orbitrap MS through an electrospray ion source operated in positive mode with a capillary voltage of 2200 V. The raw MS files were analyzed and identified using the Byonic™ MS/MS search engine for peptide and protein identification (Protein Metrics by Dotmatics). The search parameters were set as follows: the protein modifications were carbamidomethylation (*C*) (fixed) and oxidation (*M*) (variable); the enzyme specificity was set to nonspecific; the maximum missed cleavages were set to three; the precursor ion mass tolerance was set to 20 ppm, and the MS/MS tolerance was 0.02 Da. Only peptides identified using high‐confidence identification were chosen for downstream protein identification analysis.

### 2.5 Cell culture and processing

WRL‐68 cells were placed in Dulbecco's modified eagle medium containing 10% phosphate buffer saline (PBS) and 1% penicillin–streptomycin solution and cultured in a 37°C, 5% CO_2_ cell incubator. Cells that grew well in the logarithmic growth phase were chosen for further testing. The cultivated WRL‐68 cells were divided into six treatment groups as follows: control, treated with normal culture medium; oxidative stressed, treated with 20 mol/L H_2_O_2_ for 24 h; MBAPs exhibiting high antioxidant capacity after isolation, treated with 80 mg/L MBAP for 24 h; H_2_O_2_ + MBAP, pretreated with 20 mol/L H_2_O_2_ for 4 h and then treated with 80 g/L MBAP for 24 h; positive control NAC group, treated with 80 mg/L NAC for 24 h; H_2_O_2_ + NAC group, pretreated with 20 mol/L H_2_O_2_ for 4 h, followed by 80 mg/L NAC for 24 h.

### 2.6 Cell viability assay

According to the method of Sheng et al. (2022) with minor modifications, the cell viability was analyzed using 3‐(4,5‐dimethylthiazol‐2‐yl)‐2,5‐diphenyltetrazolium bromide (MTT) (Sigma‐Aldrich®, Merck KGaA) assay. The WRL‐68 cells were seeded in 96‐well plates at 4 × 10^3^ cells/well and treated with MBAP for 24 h. Then, 10 μL (0.5 mg/mL) of MTT was added to each well and incubated for 2 h at 37°C in 5% CO_2_. The supernatant was removed, and the formazan was solubilized with dimethyl sulfoxide. Absorbance was measured at 490 nm using a UV MAX kinetic microplate reader (Molecular Devices, LLC).

### 2.7 Calcein‐AM/Hoechst 32258 staining

The number of viable cells in each treatment group was evaluated using Calcein‐AM and Hoechst 32258 staining. The cells' nuclei were visualized and observed qualitatively under a microscope (Nikon Corporation) after incubation for 20 min with the Hoechst 32258 and Calcein‐AM stain. Photomicrographs were taken using a fluorescence microscope EVOS® XL Core Imaging System (Advanced Microscopy Group Inc.). All assays were performed in triplicate.

### 2.8 Detection of ROS

Each treatment group's cells were planted into 6‐well plates at 1.2 × 10^4^ cells/well and then treated with MBAP for 24 h. Staining using DHE and MitoSOX™ was used to determine changes in cellular and mitochondrial ROS levels. Nuclei were visualized using the Hoechst 32258 stain as described previously.

### 2.9 Mitochondrial depolarization assay

According to the method of Wang, Huang, et al. (2021), Wang, Xing, et al. (2021) the difference in the levels of the WRL‐68 cell mitochondrial membrane potential (ΔΨ) following the MBAP and H_2_O_2_ treatments was observed using 5,5′,6,6′‐Tetrachloro‐1,1′,3,3′‐tetraethyl‐imidacarbocyanine iodine (JC‐1) staining (Beyotime Biotechnology Inc.). Plates were inoculated with 20 mM JC‐1 for 15 min at 37°C before being rinsed with PBS. After washing with PBS, the fluorescence intensities were subjectively examined using an EVOS® XL Core Imaging System.

### 2.10 Intracellular antioxidant enzymatic activity determination

The treatment groups were cultured for 12 h using the experiment's specifications. Then, the WRL‐68 cells were seeded in 6‐well plates at a density of 3 × 10^5^ cells/well. The relative contents of CAT and SOD, and MDA and GPX were analyzed by ELISA; the experimental procedures were according to the manufacturer's instructions.

### 2.11 Western blot analysis

The cells were incubated for 12 h at 37°C and 5% CO_2_ according to the groups' various treatment conditions. Nuclear proteins were extracted from the cells of each treatment group by nuclear protein extraction kit and used as the expression assay of Nrf2, and then, proteins were extracted from the cells of each treatment group by bicinchoninic acid protein quantification kit, and their concentrations were calculated and used as the expression assay of Keap1. The cell protein was then separated by electrophoresis utilizing polyacrylamide gel at a concentration of 5–10%. The gel was applied onto a polyvinylidene difluoride membrane, which was then submerged in dried skim milk at a 5% concentration for 1 h. The membranes were treated overnight at 4°C with primary antibodies against Keap1, Nrf2, and β‐actin (dilution ratio 1:500), followed by five washes with tris‐buffered saline with Tween® (Sigma‐Aldrich®, Merck KGaA). The membranes were then treated at room temperature for 2 h with a secondary antibody (1:10,000 dilution). The blots were examined using a chemiluminescence detection system (GE Healthcare Life Sciences). The Western blot data were processed utilizing ImageJ2x 2.1.4.7 software (Rawak Software, Inc.).

### 2.12 Statistical analysis

The obtained data are the averages of six experiments. The data were analyzed using SPSS® (Statistical Software™ Version 19.0, IBM SPSS Statistics), and the significant differences between the averages (*p* < .05) were checked using Tukey's honestly significant difference test for multiple comparisons. Graphs were plotted with SigmaPlot 13.0 software (Systat Software Inc. https://sigmaplot.software.informer.com/13.0/).

## 3 RESULTS AND DISCUSSION

### 3.1 Isolation and purification of the MBPH

**FIGURE 1:**
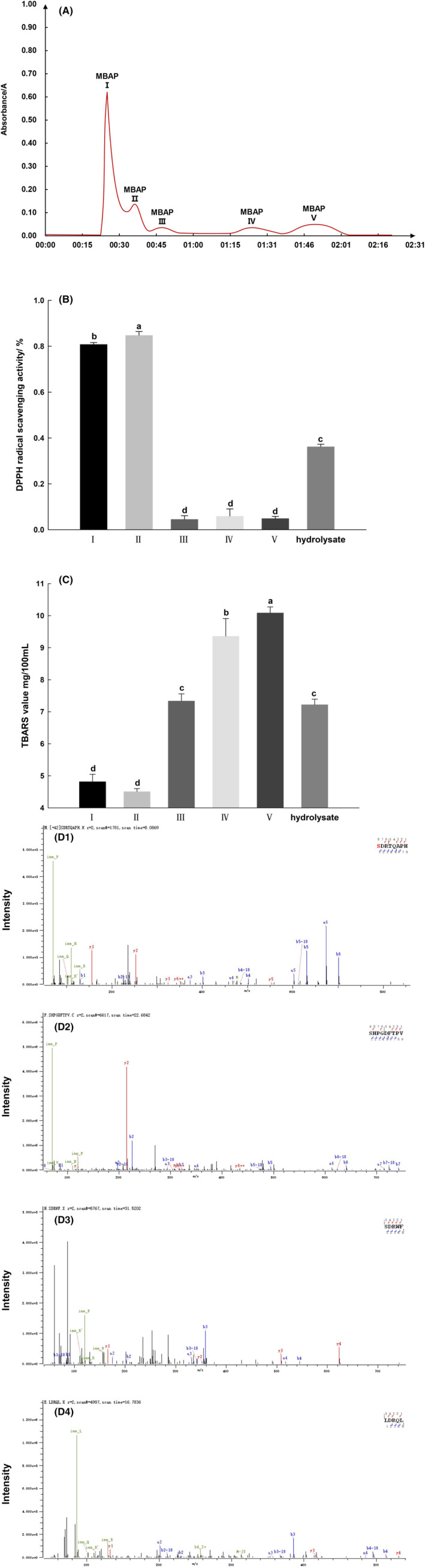
(A) Dextran gel SephadexG‐15 chromatogram shows 5 peaks from left to right and an elution time of approximately 2 h. (B) The ability of five peptides to scavenge DPPH radicals after extraction and purification and the TBARS value (C), and the mass spectrum of the RP‐HPLC chromatograms second‐grade mung bean peptide sequences (D1) SDRTQAPH, (D2) SHPGDFTPV, (D3) SDRWF, and (D4) LDRQL by LC–MS/MS using a Q Exactive Hybrid Quadrupole‐Orbitrap mass spectrometer. Significant differences between each peptide and control are indicated as (a–d), (*p* < .05). Error bars represent the standard deviations of three measurements.

The MBPH was processed for isolation and purification of MBAPs with a high antioxidant activity using a Sephadex G‐15 chromatographic column. As shown in Figure 1A, the MBPH was divided into five MBAP fractions designated as I, II, III, IV, and V. The antioxidant activities of these five fractions were significantly different at the same concentration (Figure 1B), in which the DPPH radical scavenging capacity of the MBPH‐II fraction reached 84.82%, remarkably higher than that of the other fractions. The TBARS value reflected the malondialdehyde content in the system, and a low TBARS value indicated less oxidization. The TBARS value of MBPH‐II was 4.5 mg/100 mL, which is significantly lower compared to other fractions (Figure 1C).

The average relative molecular weight of this second fraction of MBAPs was about 870 Da, as deduced from the peak time of molecular weight standards, which is consistent with the findings of Ye (2021). Several studies have confirmed that the smaller the molecular weight of a protein hydrolysate, the stronger its antioxidant capacity. Furthermore, the protein hydrolysates with higher antioxidant capacities are mostly below 1000 Da in molecular weight. Therefore, the second fraction of MBAPs was expected to have higher antioxidant activity. Senphan and Benjakul (2014) found that the higher antioxidant capacities and improved scavenging activity of lower molecular weight peptides, such as MBAPs, result because they have less spatial site resistance and bind to free radicals to form more stable products, thus blocking their chain reactions (Wen, 2021). However, when the molecular weight is too small, an active polymeric structure cannot be formed in the peptide segment (Guan et al., 2022), which makes it difficult to encapsulate the lipid droplets (Diao, 2019) and affects its antioxidant capacity.

### 3.2 Sequence analysis of the MBAP fractions

To identify the amino acid composition and sequence of the MBAP II fractions, LC–MS/MS was used for analysis, and a search was performed using the Byonic™ database to obtain the MBAP fractions sequences. Figure 1D shows that the second fraction of MBAPs contained four main peptides with molecular weights ranging from about 644 to 954 Da. The amino acid sequences of the peptides were as follows: Ser‐Asp‐Arg‐Thr‐Gln‐Ala‐Pro‐His (Figure 1D1), Ser‐His‐Pro‐Gly‐Asp‐Phe‐Thr‐Pro‐Val (Figure 1D2), Ser‐Asp‐Arg‐Trp‐Phe (Figure 1D3), and Leu‐Asp‐Arg‐Gln‐Leu (Figure 1D4).

The molecular weights of the MBAPs were consistent with the findings that the molecular weights ranged from about 500–1800 Da. Several studies have found that protein hydrolysates exhibiting antioxidant capacity have molecular weights less than 1000 Da, such as potato protein hydrolysate (280–800 Da; Cheng et al., 2010), purple date (*Zizyphus jujuba*) protein hydrolysate (678.36–482.27 Da; Memarpoor‐Yazdi et al., 2013), hemp protein hydrolysate (441.0–924.5 Da; Lu et al., 2010), and chickpea protein hydrolysate (717.4 Da; Zhang et al., 2011); these results are consistent with the present study.

The amino acids in the sequence of a protein peptide are directly related to its antioxidant activity. For example, the aromatic amino acid Phe, and the imidazole‐containing amino acid His, can be the active sites of antioxidant peptides (Zhang et al., 2013). High content of hydrophobic amino acids is also a common feature of antioxidant peptides (Ajibola et al., 2011). The MBAPs peptide sequence results showed that they were rich in hydrophobic amino acids and aromatic amino acids; the percentages of hydrophobic amino acids in the peptides were 37.5%, 55.6%, 40%, and 40%, respectively, to MBAP II. Furthermore, this study found that most of the MBAP sequences contained Pro; Trp and Pro are suggested to be important to the activity of antioxidant peptides (Hernández‐Ledesma et al., 2005). Additionally, Saiga et al. (2003) demonstrated that all porcine myogenic fiber antioxidant peptide sequences contain Asp. Therefore, we speculated that the MBAPs' excellent 1,1‐diphenyl‐2‐picryl‐hydrazyl radical scavenging and lipid peroxidation inhibiting ability could be related to the presence of His, and hydrophobic amino acids, such as Trp, Asp, and Pro, in the peptide sequences.

### 3.3 Effect of MBAPs on WRL‐68 cell viability

**FIGURE 2:**
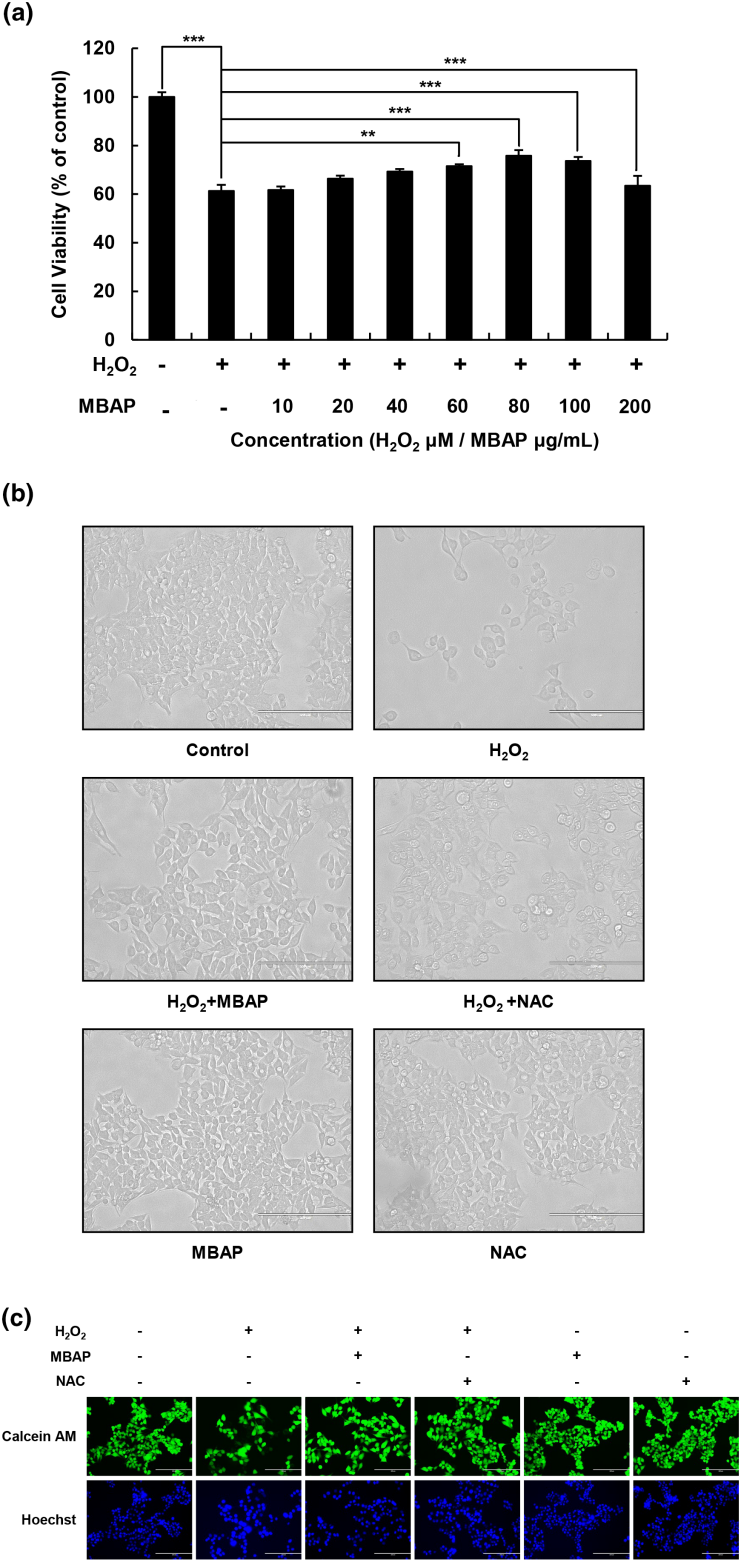
WRL‐68 cell viability determined by MTT assay and photomicrograph. (a) Cells were treated with H_2_O_2_ (0, 20 μM) for 4 h and then treated with MBAP (0, 10, 20, 40, 80, 100, 200 μg/mL) for 24 h. Error bars represent the standard deviations of three measurements. (b) Photomicrographs of samples (a) were viewed by an optical microscope (100×). scale bars = 200 μm. Values are represented as the means ± SD (*n* = 5). **p < .01, ***p < .001 versus control group (treated with 20 μM H_2_O_2_). (c) Effect of MBAP on H_2_O_2_‐induced oxidative stress in a WRL‐68 cell model of live cells (Calcein AM/Hoechst staining). scale bars = 200 μm.

WRL‐68 cells are widely used in toxicology studies because this cell line exhibits morphological, functional, and cytoskeletal characteristics similar to those of primary hepatocytes in culture (Ramirez et al., 2000). The effect of MBAPs (10–200 μM) on oxidation‐induced cell viability of the WRL‐68 cells was analyzed by the MTT assay (Figure 2a). The H_2_O_2_‐induced WRL‐68 cells' viability decreased greatly by about 40% compared with the control group. However, the viability of oxidation‐induced cells improved with increased MBAP concentration in the range of 10–100 μg/mL. At 80 μM, viability reached 80%, which was markedly higher than that of the H_2_O_2_‐induced treatment group; this indicated that MBAPs could improve the oxidation‐induced cell damage and increase the cell survival rate. However, the effect on cell viability appeared to be saturated at 80 μg/mL. Furthermore, when the MBAP concentration exceeded 100 g/mL, the cell viability decreased slightly but was still better than the H_2_O_2_‐induced treatment group. Cell viability provides a visual representation of the effect of an active agent on viable cells (Yadav et al., 2022). By analyzing the WRL‐68 cells' viability, the protective effect of MBAPs on oxidation‐induced cells was confirmed.

### 3.4 Effect of MBAPs on WRL‐68 cell morphology

The effect of MBAP treatment on oxidation‐induced WRL‐68 cell morphology and viability is shown in Figure 2b,c. The fluorescence microscopy images show that there were considerable differences in the morphology of oxidation‐induced cells between the control, H_2_O_2_‐induced, and MBAP‐treated oxidation‐induced groups (Figure 2b). NAC is an antioxidant composed of amino acids, and it is an antioxidant approved by the Food and Drug Administration in the United States (Rhodes & Braakhuis, 2017). MBAP is similar in composition to it and exhibits the same effects as NAC.

The cells in the control and MBAP‐treated groups were intact with a normal structure; the cells in the H_2_O_2_‐induced group were larger, and the cell colonies were sparse. The addition of NAC resulted in sharper cell edges and a more uniform distribution of nuclei in oxidatively stressed cells, and an increase in cell number was clearly seen compared to the model group. However, this situation improved markedly after MBAP and NAC treatment; the cell numbers increased, and the cell size improved tremendously and was similar to the control group. Notably, MBAP showed superior ameliorations in cell morphology and number than NAC.

It has been demonstrated that ROS can occasionally be produced in cells due to structural alterations in deoxyribonucleic acid; this can harm cells' health and functionality (Sifuentes‐Franco et al., 2018). Calcein‐AM/Hoechst 33258 was used to stain the WRL‐68 cells to evaluate the viability of the MBAP‐treated oxidation‐induced cells (Figure 2c). Calcein‐AM is a cell‐permeant dye that is changed to a brilliant yellow‐green fluorescent color by the intracellular esterases of viable cells. As shown in Figure 2c, Calcein‐AM was uniformly concentrated in the cytoplasm of the viable cells in the control group. In the H_2_O_2_‐induced treatment group, Calcein‐AM showed separation compared with the control group, indicating fewer viable cells in this group. Cells in the NAC intervention oxidation‐induced group showed uniform and concentrated yellow‐green fluorescence under fluorescence microscopy compared to the H_2_O_2_ group, indicating that the number of surviving cells was higher in this group. The results showed that Calcein‐AM in the MBAP‐induced treatment group was also uniformly condensed in the cytoplasm of viable cells, similar to the NAC‐treated group, with significant differences visible in comparison with the H_2_O_2_‐treated group (Figure 2c).

Hoechst 32258 binds to cellular DNA directly and emits blue fluorescence when exposed to UV light. As shown in Figure 2c, the nuclei of the control group were uniformly stained with bright blue fluorescence; furthermore, they were smaller than the nuclei of the H_2_O_2_‐treated group, which were larger with blurred edges. Compared with the H_2_O_2_‐induced cells, the nuclei of the MBAP‐treated oxidation‐induced treatment group were substantially smaller in size and had remarkably improved edges, this result was consistent with the NAC‐treated group. According to these results, the MBAPs substantially increased the vitality of the WRL‐68 cells that had undergone oxidation.

### 3.5 Effect of MBAPs on intracellular ROS, mitochondrial oxidative damage, and ΔΨ of WRL‐68 cells

**FIGURE 3:**
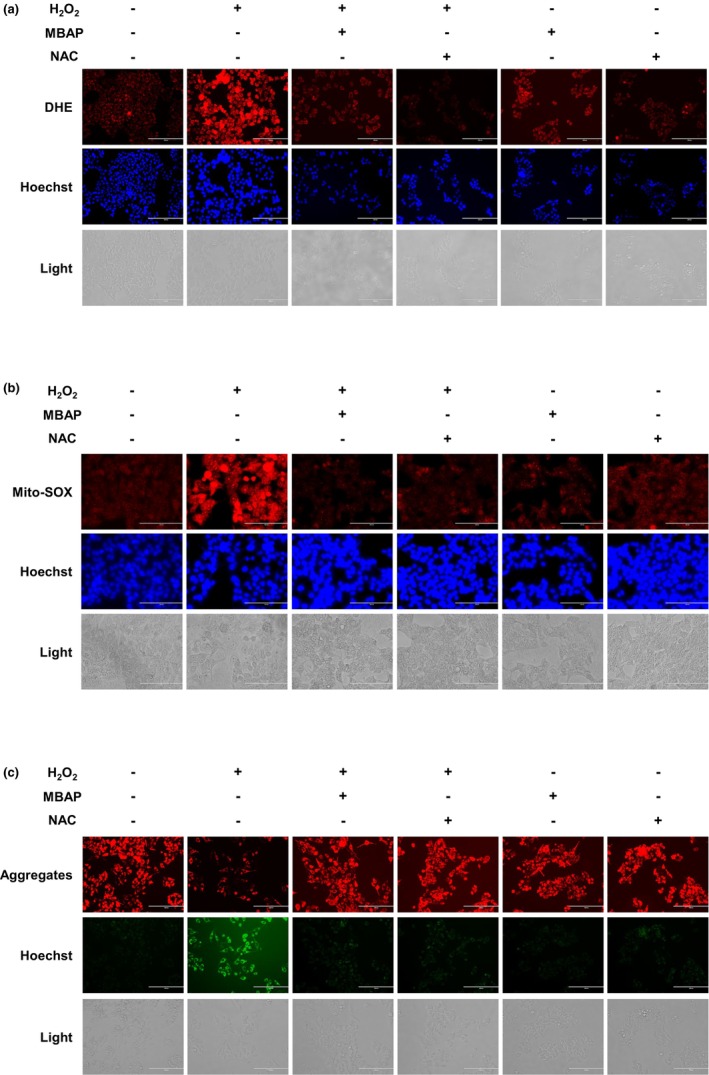
Effect of MBAP on H_2_O_2_‐induced oxidative stress in a WRL‐68 cell model of (a) intracellular ROS, (b) mitochondrial oxidative damage, and (c) mitochondrial membrane potential.

The production, utilization, and elimination of intracellular ROS are dynamically balanced under normal conditions (Chen et al., 2020). Excessive ROS generation is considered a sign of oxidative stress, which causes oxidative damage to cells and can cause mitochondrial dysfunction inducing cell death (Betteridge, 2000). The effects of MBAPs on intracellular ROS, mitochondrial ROS, and ΔΨ were analyzed in H_2_O_2_‐induced WRL‐68 cells to evaluate their function in regulating cellular oxidative stress levels. As shown in Figure 3, the fluorescence intensity of cells stained by the DHE and Hoechst 32258 stains was enhanced in the H_2_O_2_‐induced WLR‐68 cells, indicating increased ROS production. In contrast, the fluorescence intensity of oxidation‐induced cells was significantly reduced by MBAP and NAC treatments, indicating that ROS production was dramatically inhibited. And the ROS production inhibition was stronger in the MBAP‐treated oxidation‐induced group compared with the NAC‐treated H_2_O_2_‐induced group, and the relative fluorescence intensity decreased to a level close to that of normal cells (Figure 3), indicating that the MBAPs maintained the endostasis of the H_2_O_2_‐induced cells. Levels of ROS in the MBAP‐treated cells were not substantially different from that of the control group (Figure 3a).

Mitochondrial dysfunction is the primary factor causing oxidative stress‐induced cell death (Liu et al., 2008). Therefore, cellular mitochondrial ROS (using MitoSOX™ staining) and ΔΨ (using JC‐1 staining) were determined. MitoSOX™ dye penetrates living cells, selectively targets oxidized mitochondria, and produces intense red fluorescence. There was increased red fluorescence in the H_2_O_2_‐induced cell treatment group compared with the control group (Figure 3b). The red fluorescence, corresponding to the cellular mitochondrial ROS, of the MABP‐treated oxidation‐induced cell group underwent a pronounced ablation and was notably different from that of the H_2_O_2_‐induced cell group. Notably, the quenched red fluorescence of MBAP on oxidation‐induced cells was significantly better than that of NAC, suggesting that MBAP exhibited a better inhibitory effect on H_2_O_2_‐induced oxidative damage of mitochondria.

The production of intracellular ROS leads to the loss of ΔΨ; when the value of ΔΨ is low, the JC‐1 stain present in the mitochondrial matrix as a monomer produces green fluorescence. As shown in Figure 3c, the ΔΨ results were consistent with the intracellular and mitochondrial ROS results. Red fluorescence, corresponding to cellular mitochondrial ROS, increased in the MBAP‐induced oxidized cell group, while green fluorescence, corresponding to the ΔΨ, was enhanced in the H_2_O_2_‐induced cell group; these results indicated that the MBAPs inhibited oxidation‐induced mitochondrial damage in the cells. Furthermore, similar to the results of Mito‐SOX, the enhancement of ΔΨ in oxidatively stressed cells by MBAPs was superior to that of NAC, as evidenced by the lower intensity of green fluorescence after the addition of MBAPs to oxidatively stressed cells than that under the intervention of NAC. These results suggest that MBAPs possess potent antioxidant properties and can improve intracellular ROS levels to maintain normal cellular metabolism.

### 3.6 Effect of MBAPs on antioxidant enzyme activity and MDA content of WRL‐68 cells

An imbalance between free radicals and the body's antioxidant defense systems can be affected by oxidative stress (Jiang et al., 2016), which can damage cell membranes and cause cellular damage. However, it can be ameliorated by the activity of antioxidant enzymes, including CAT, SOD, and GSH. These enzymes are widely present in the body and can eliminate ROS production and maintain the dynamic balance (Gou et al., 2020; Liang et al., 2021). In this study, MDA was used as a marker of oxidative stress.

**FIGURE 4:**
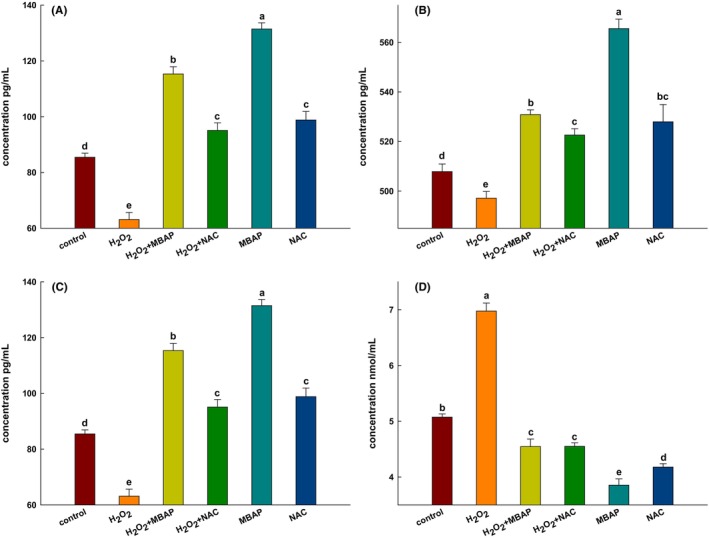
Effect of MBAP on the content of (A) SOD, the relative activity of (B) GPX, the content of (C) CAT, and (D) MDA in WRL‐68 cells under oxidative stress. Significant differences between each group and control are indicated as (a–d). Significant differences between each group and control are indicated as (a–d), (*p* < .05). Error bars represent the standard deviations of three measurements.

The results indicate that SOD activity was substantially reduced in H_2_O_2_‐treated WRL‐68 cells (Figure 4); however, its activity in the MBAP‐treated oxidation‐induced group was significantly reversed and significantly higher than that in the control cell group (*p* < .05; Figure 4A). In addition, the SOD levels in the MBAP‐treated cell group were markedly higher than those of the other treatment groups, and even 20 ng/mL higher than that in the NAC‐treated group.

In addition, GPx, an essential low molecular scavenger in mammalian cells, can be used to evaluate the antioxidant capacity of cells. This enzyme plays an important role in cellular metabolism, including protecting cells from the harmful effects of ROS (Li et al., 2022). Compared with the H_2_O_2_ oxidation‐induced group, the GPX levels increased approximately 1.6‐fold in the MBAP‐treated oxidation‐induced WRL‐68 cells, demonstrating a potential protective effect on oxidatively damaged cells (Figure 4B), and a similar situation was observed in the NAC‐treated model group, but it was a considerably less effective in promoting GPX production than the MBAP‐treated model group.

Another essential enzyme of the biodefense system is CAT, a marker enzyme of the peroxisome, which scavenges H_2_O_2_ protecting cells from its toxicity (Barteková et al., 2021). Compared with the control group, the CAT level was greatly increased in the MBAP‐treated oxidation‐induced group; this result was consistent with those of the SOD and GPX levels in the oxidatively induced cell group by NAC intervention (Figure 4C).

In addition, the MDA levels in the MBAP‐treated oxidation‐induced group were reduced by about 1.5‐fold compared with the H_2_O_2_ oxidation‐induced group; the MDA levels in the MBAP‐treated cells were also approximately 2‐fold lower than those in the control group cells, and around 1.1‐fold lower than those of NAC‐treated group, indicating that MBAPs protect the normal metabolism of oxidatively damaged cells by increasing SOD activity and decreasing MDA levels (Figure 4D).

In conclusion, MBAPs reversed the depletion of the total antioxidant status of oxidation‐induced cells by restoring SOD, CAT, and GPX activity. Furthermore, MBAPs reduced the MDA content in oxidation‐induced cells with no adverse effects on the cells.

### 3.7 Effect of MBAPs on WRL‐68 cells' relative protein expression levels of Keap1 and Nrf2

The Keap1‐Nrf2 signaling pathway governs antioxidant enzymes, such as GPX and SOD, and plays a pivotal role in antioxidation by converting peroxides to less harmful or innocuous molecules (Kumar et al., 2022). The antioxidant impact produced by activating the Keap1‐Nrf2 signaling pathway and its downstream genes in normal cells is an important defensive mechanism to withstand foreign chemicals and suppress cancer. The Nrf2 factor is a transcriptional regulator of the body's antioxidant stress response; Keap1 forms part of an E3 ubiquitin ligase that regulates Nrf2 activity. Therefore, Keap1 inhibits Nrf2 activity under normal conditions.

Several studies have demonstrated that Nrf2 dissociates from Keap1 in oxidation‐induced cells, allowing it to enter the nucleus and activate target genes associated with androgen response elements (Baird & Yamamoto, 2020). This activity includes initiating antioxidant response elements, regulating the expression of phase II detoxification and antioxidant enzyme genes, and increasing cellular resistance to oxidative stress responses (Qu et al., 2014; Ruwali & Shukla, 2021; Yamamoto et al., 2018). By initiating the transcription of downstream antioxidant proteins and enzymes, such as SOD and GPX, Nrf2 enhances the cellular antioxidant state (Lin et al., 2019).

**FIGURE 5:**
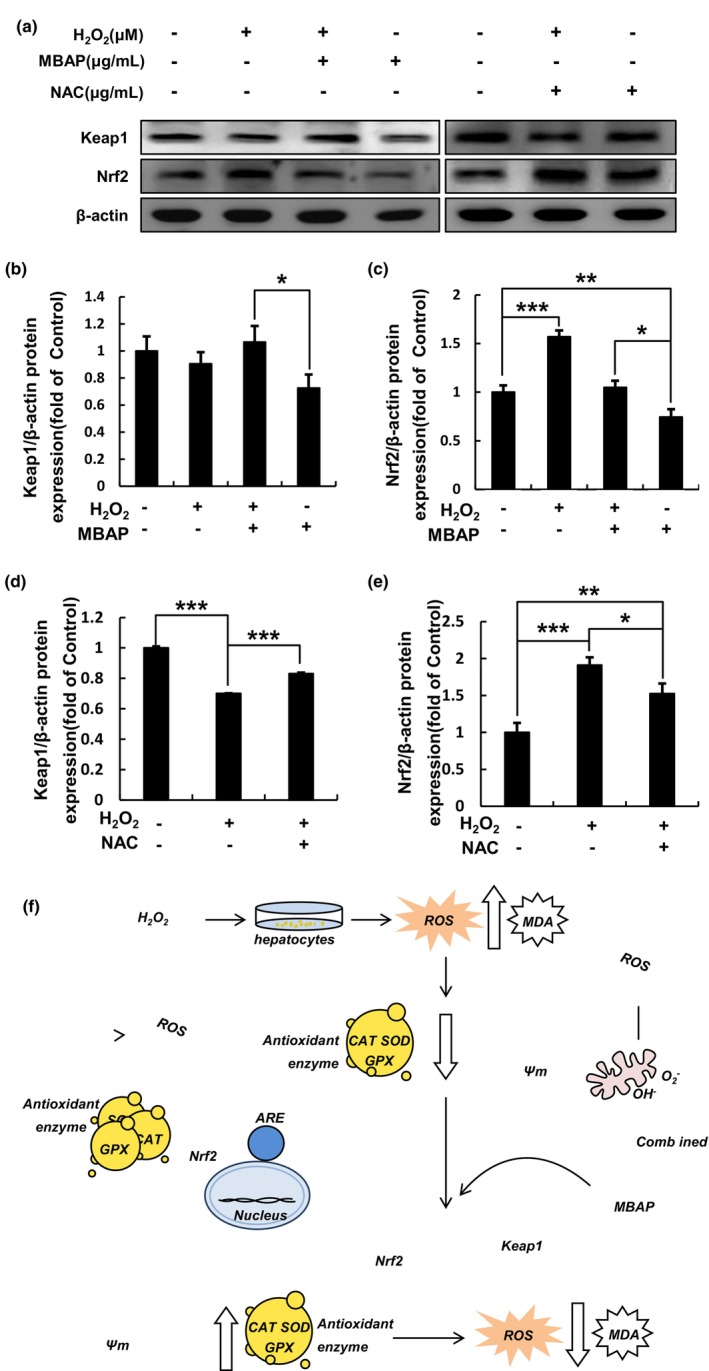
Effect of MBAP on the expression levels of Keap1 and Nrf2 proteins in WRL‐68 cells under oxidative stress by Western blotting analysis. (a) Keap1 and Nrf2 phosphorylation was assessed by Western blotting analyses. Relative protein levels of (b), (d) Keap1 and (c), (e) Nrf2 are shown in (a). Significant differences in Keap1 and Nrf2 expression levels between the treated groups and the control group are indicated by *. *p < .05, **p < .01, ***p < .001. Error bars represent the standard deviations of three measurements. (f) Possible antioxidant mechanism of MBAP.

As shown in Figure 5, the H_2_O_2_‐induced group exhibited significantly up‐regulated Nrf2 and markedly inhibited Keap1 expression compared with the control group; this result was dramatically altered in the NAC‐treated oxidation‐induced group, which displayed down‐regulated Nrf2 (Figure 5d) and up‐regulated Keap1 (Figure 5e) expression compared with the H_2_O_2_‐treated group. The results showed that NAC exhibited a direct Scavenging role against ROS, indicating that NAC inhibited oxidative stress in oxidation‐induced cells and restarted the ubiquitination of Nrf2 by Keap1. Compared with the H_2_O_2_‐treated group, the MBAP oxidation‐induced group exhibited a down‐regulation of intranuclear Nrf2 expression (Figure 5c) and an up‐regulation of Keap1 expression (Figure 5b). And the expression level of intranuclear Nrf2 in the oxidation‐induced cell group by MBAP treatment was close to that of the control group, which again indicated that MBAP could prevent the cells from being attacked by ROS by promoting the expression of intranuclear Nrf2 to produce antioxidant enzymes, such as SOD, GPX, thus maintaining the cellular redox homeostasis.

These results differ from previous studies' findings; this may be related to the degree of activation of the oxidative stress response and the reduction of oxygen‐free radical production by oxidative enzymes, which is consistent with the findings of Yang et al. (2021).

### 3.8 Antioxidant mechanism of MBAP on H_2_O_2_ ‐induced WRL‐68 cells

The oxidation‐induced redox homeostasis experiment of MBAP on WRL‐68 revealed that MBAP has a strong antioxidant capacity, and its antioxidant mechanism is shown in Figure 5f. The antioxidant activity of MBAPs is determined by its amino acid sequences. O_2_ is mainly consumed in mitochondria, and electron‐bearing products including O_2_^−^, H_2_O_2_, OH^−^, NO, etc. are generated in the mitochondrial electron transport chain. These electron‐bearing products are involved in energy metabolism on the one hand, and generate oxidation products such as ROS, reactive nitrogen species, and lipid peroxides through a series of reactions on the other hand (Gao et al., 2021). The mitochondrial membrane potential controls the rate of ROS production by mitochondria, and when mitochondria are exposed to outside stimuli such as H_2_O_2_ and hyperlipidemia, the production of reactive oxygen species in the body rises dramatically (Bravo‐Sagua et al., 2013).

In this investigation, we discovered that MBAP was able to scavenge significant amounts of free radicals in vitro. This finding was supported by the high DPPH scavenging rate, low TBARS values, and mitochondrial staining, which showed that MBAP raised the membrane potential in cells under oxidative stress and removed significant amounts of ROS from the inner mitochondrial membrane. Since the cationic groups in its amino acid sequence may attach to free radicals like O_2_^−^ and OH^−^ (Zhang, Li, et al., 2022; Zhang, Zhao, et al., 2022), MBAP can directly scavenge free radicals, lowering the production of ROS.

In addition, the Keap1/Nrf2 signaling pathway is an important pathway in response to oxidative stress, the conformation of the ubiquitinases involved in Keap1 changes and the amino acid residues on Nrf2 are unable to capture the normal information, resulting in the inability of Nrf2 to be ubiquitinated, and therefore, the level of Nrf2 protein is elevated at this time (Baird et al., 2013).

Because of that, Nrf2 can move easily to the nucleus, where it binds to the ARE and activates several enzymes, including antioxidant and phase II detoxification enzymes that are downstream of transcription (Hirotsu et al., 2012). Western blot results n H_2_O_2_‐induced WRL‐68 cells effectively explained the antioxidant mechanism of MBAP. These results showed a decrease in Nrf2 expression and an increase in Keap1 expression of MBAP‐treated oxidative stressed cells, and this change in expression was similar to the control group, indicating that MBAP effectively inhibited oxidative stress by binding free radicals to levels that were close to normal. Along with oxidation‐induced intracellular Nrf2 expression was higher after MBAP intervention than in the control group, suggesting that MBAP promotes Nrf2 expression besides direct scavenging of ROS and drives downstream antioxidant enzyme genes.

In summary, the antioxidant mechanism of MBAP is to stimulate the Keap1/Nrf2 signaling pathway and promote transcription of downstream antioxidant enzyme genes while binding free radicals through amino acid sequences to scavenge excess ROS in cells and protect it from oxidative damage.

## 4 CONCLUSION

This study's results demonstrated that oxidation‐induced hepatocyte injury could be effectively alleviated by treatment with MBAPs. Oxidation‐induced hepatocyte injury was established to be mainly related to the activation of the Keap1/Nrf2 signaling pathway; this was demonstrated by the restored expression of Keap1 and Nrf2 in MBAP‐treated H_2_O_2_‐induced cells and the increased levels of antioxidant enzymes and suppressed levels of MDA and free radicals. This study comprehensively evaluated the effects of MBAPs on oxidation‐induced hepatocytes to provide a basis for further elucidation of their role in regulating oxidation‐induced abnormal liver metabolism. The following research will focus on the mechanism of action of MBAP in regulating the abnormal metabolism of the liver to provide a technical basis for the high‐value utilization of mung bean protein.

## AUTHOR CONTRIBUTIONS

**Xue Miao:** Investigation (equal); methodology (equal); software (equal); validation (equal); writing – original draft (equal). **Xin Liu:** Investigation (equal); methodology (equal); validation (equal). **Hongsheng Chen:** Supervision (equal); writing – review and editing (equal). **Changyuan Wang:** Funding acquisition (equal); project administration (equal); supervision (equal). **Jingjing Diao:** Conceptualization (equal); funding acquisition (equal); project administration (equal); writing – review and editing (equal).

## FUNDING INFORMATION

This work was supported by the Central Guidance on Local Science and Technology Development Fund [grant number DQKJJYD0001]; the Natural Science Foundation of Heilongjiang Province of China [grant number SS2022C002]; the Heilongjiang Major Science and Technology Projects [grant number 2021ZX12B05]; and the Heilongjiang Postdoctoral Fund [grant number LBH‐Z20206].

## CONFLICT OF INTEREST STATEMENT

The authors declare that they do not have any conflict of interest.

## ETHICS STATEMENT

Ethical Review: “This study does not involve any human or animal testing.”

Informed Consent: Written informed consent was obtained from all study participants.

## Data Availability

All data generated or analyzed in this study are included in this published article.
